# Infections with Immunogenic Trypanosomes Reduce Tsetse Reproductive Fitness: Potential Impact of Different Parasite Strains on Vector Population Structure

**DOI:** 10.1371/journal.pntd.0000192

**Published:** 2008-03-12

**Authors:** Changyun Hu, Rita V. M. Rio, Jan Medlock, Lee R. Haines, Dana Nayduch, Amy F. Savage, Nurper Guz, Geoffrey M. Attardo, Terry W. Pearson, Alison P. Galvani, Serap Aksoy

**Affiliations:** 1 Department of Epidemiology and Public Health, Yale University School of Medicine, New Haven, Connecticut, United States of America; 2 Department of Biochemistry and Microbiology, Petch Building, University of Victoria, Victoria, British Columbia, Canada; Institute of Tropical Medicine, Belgium

## Abstract

The parasite *Trypanosoma brucei rhodesiense* and its insect vector *Glossina morsitans morsitans* were used to evaluate the effect of parasite clearance (resistance) as well as the cost of midgut infections on tsetse host fitness. Tsetse flies are viviparous and have a low reproductive capacity, giving birth to only 6–8 progeny during their lifetime. Thus, small perturbations to their reproductive fitness can have a major impact on population densities. We measured the fecundity (number of larval progeny deposited) and mortality in parasite-resistant tsetse females and untreated controls and found no differences. There was, however, a typanosome-specific impact on midgut infections. Infections with an immunogenic parasite line that resulted in prolonged activation of the tsetse immune system delayed intrauterine larval development resulting in the production of fewer progeny over the fly's lifetime. In contrast, parasitism with a second line that failed to activate the immune system did not impose a fecundity cost. Coinfections favored the establishment of the immunogenic parasites in the midgut. We show that a decrease in the synthesis of *Glossina* Milk gland protein (*GmmMgp*), a major female accessory gland protein associated with larvagenesis, likely contributed to the reproductive lag observed in infected flies. Mathematical analysis of our empirical results indicated that infection with the immunogenic trypanosomes reduced tsetse fecundity by 30% relative to infections with the non-immunogenic strain. We estimate that a moderate infection prevalence of about 26% with immunogenic parasites has the potential to reduce tsetse populations. Potential repercussions for vector population growth, parasite–host coevolution, and disease prevalence are discussed.

## Author Summary

In many cases, parasites adapt to their hosts' biology over time and the extent of their harmful effects gradually diminishes. Insect-transmitted parasites such as African trypanosomes, however, are unusually pathogenic for their mammalian hosts because they rely on their invertebrate hosts for transmission to the next mammalian host. To ensure their maximum transmission, it is essential that parasite infections do not compromise insect host's fitness traits, including longevity and host-finding ability. Our results in tsetse indicate that, as theory predicts, trypanosome infections do not reduce host longevity. Instead, they divert host resources from reproduction and can reduce reproductive output by as much as 30%. Such loss of reproductive fitness occurs as a result of the induction of tsetse's immune responses. A closely related non-immunogenic parasite line does not induce host responses and does not compromise host fecundity. It is possible that host immune responses are needed in the case of the immunogenic line to control the parasite density to prevent excessive host damage. Because tsetse are viviparous and each adult female typically gives rise to only few progeny during their lifetime, even modest costs on reproduction can have a significant impact on host abundance. Our model predicts that if the prevalence of immunogenic parasite infections in tsetse populations reaches over 26%, they begin to have a negative impact on population growth rate. Infection rates as high as 30% have been reported with trypanosomes in the field. Our laboratory findings coupled with our modeling studies now provide a framework to investigate the status of co-infections, host immune activation processes, fecundity outcomes, transmission dynamics, and host virulence phenotypes in natural tsetse–trypanosome populations.

## Introduction

Insect vectors are essential for the transmission of malaria and African sleeping sickness, among many other diseases. Despite the high disease incidence in mammalian hosts, infection prevalence in insect vectors is typically low. For example, with the tsetse vectors of trypanosomes that cause African sleeping sickness, often only 1–3% of flies are infected in field populations (reviewed in [1]). This is also reflected in laboratory experiments where although all flies are subjected to an infectious bloodmeal, only a few show established midgut infections [2],[3]. Successful parasite infection of vectors likely reflects a balance between the effectiveness of the vector insect's immune response and the ability of the parasite to evade this response. The ability to resist parasitism has been shown to carry a fitness cost [4]–[7]. In several cases, a decrease in reproductive output has been shown to result from activation of the host's immune responses [8]–[10]. In a recent study, transgenic mosquitoes which expressed molecules that conferred parasite resistance were found to be more fit than wild type insects when exposed to malaria parasites [11]. Presumably the transgenic insects were able to eliminate infections prior to the activation of costly natural immune responses. In parasitized insects, the parasites likely compete for the insect's restricted nutritional resources. Here a balance also probably exists, since reductions in host survival or fitness would diminish the likelihood of parasite transmission. To compensate for nutrient competition while maintaining longevity, many insect vectors exhibit reduced or delayed reproduction as a trade-off during parasite infection (reviewed in [10]). Understanding the effect of parasitism on vector fitness is fundamental to predicting the future trajectory of coevolution between parasites and their hosts and eventual disease transmission dynamics.

Little has been reported on the impact of trypanosome infection outcomes on tsetse. Unlike most insects that are oviparous, tsetse females are viviparous producing one larva at a time, a process that results in few offspring produced over a lifespan. The pregnant females nurture their single larva *in utero* via specialized accessory gland (milk gland) secretions. It is likely that tsetse's viviparous character would result in different outcomes on lifetime fitness traits when compared to other insects where reproductive output is greatest and most sensitive to adverse occurrences in early adulthood [12]. In addition to reproductive fitness, vector longevity is especially relevant as African trypanosomes undergo multiple stages of differentiation in tsetse and require extensive developmental periods before transmission to their next mammalian host.

During blood-feeding, adult tsetse can acquire bloodstream form (BSF) trypanosomes from mammalian hosts. During the early course of infection, BSF, which differentiate and replicate as procyclic forms in the midgut, undergo a massive attrition. In a small percentage of flies, parasites continue to proliferate and establish midgut infections. The mechanisms involved in parasite elimination are likely based on tsetse immune responses [13] and may include lectin agglutination [14],[15], other lectin-like activities [16],[17], antioxidant activity [18]–[20] and killing by antimicrobial peptides (AMPs) [2],[18]. Both AMP transcripts and their encoded products are produced during parasite attrition in the midgut [2],[18],[21]. Additional support for a role of AMPs in resistance comes from studies where knock down of AMP expression in tsetse resulted in increased parasite prevalence *in vivo* [3] and where recombinant Attacin has been shown to exhibit trypanolytic activity *in vivo* [22].

In this study, we examined the cost to tsetse of resistance to trypanosome infections. We examined whether *T. b. rhodesiense* midgut infections can increase mortality and reduce reproductive fitness and cause delayed life-history effects on future progeny. We evaluated the cost of infections using two parasite lines, which differ in their ability to activate tsetse immune responses. We identified and discuss one putative mechanism by which tsetse reproductive physiology might be compromised upon parasite infection. Using a mathematical model, we then estimated the impact of fecundity cost on tsetse populations and discuss the putative effect of this cost on disease transmission.

## Materials and Methods

### Parasite and insect species

BSF of the YTat1.1 parasites were derived from *T. brucei rhodesiense* stabilate TREU 164 (TREU = Trypanosomiasis Research Edinburgh University). TREU 164 represents passage 21 in mice from parasites originating from a capsule of bovine blood on which *Glossina pallidipes* captured at Lugala, Busoga, Uganda, in 1960, were allowed to feed. The pedigree of TREU 164 has been published [23]. The ETat3 variant derived from TREU 164 was triply cloned at Yale and referred to as YTat1. ETat3, and similarly, YTat1 clone1 (YTat1.1) are both highly virulent to the mammalian host, in that mice, even if infected with only a single organism, invariably die during the first parasitic wave. Procyclic culture forms (PCF) of YTat1.1^WT^ cells were derived from BSF taken from infected rat blood and maintained axenically at 28°C in SDM-79 medium supplemented with 10% heat inactivated fetal bovine serum and penicillin-streptomycin antibiotic cocktail [24].

For YTat1.1^EP^ selection, YTat1.1^WT^ PCF were grown to about 8×10^7^ cells/ml and diluted with fresh medium (1:20) once a week. The selection of the YTat1.1^EP^ phenotype of trypanosomes was reproducible in three different experiments and was observed after maintaining cells under high-density culture conditions for at least two months. For maintenance of YTat1.1^WT^ and the selected YTat1.1^EP^ line, parasite cultures were split three times per week, after they typically reached 6–8×10^6^ cells/ml. The YTat1.1^WT^ cells (PCFs or the BSFs) are not able to establish salivary gland infections in the fly.

### Fly infections

*Glossina morsitans morsitans* (Westwood) flies were maintained in the insectary at Yale University [25]. To initiate infections, newly emerged teneral flies were given a blood meal containing PCFs (10^5^ cells/ml). Parasite infection prevalence in flies was confirmed by midgut dissection and microscopic viewing at designated times. Three independent experiments were performed. No significant difference was seen by arcsine transformation analysis, which allowed pooling of the groups. Chi-square analysis was used on the pooled data.

### Northern blot analysis of tsetse immune responses

Fat body was dissected from individual trypanosome-infected or uninfected flies and homogenized in Trizol (Invitrogen, Carlsbad, CA) to extract total RNA following the manufacturer's instructions. Total RNA (10 µg per sample) was analyzed on a 1.5% agarose gel and UV crosslinked onto nylon membrane (Hybond N+, Amersham Pharmacia Biotech, NY). The membrane was hybridized with P^32^-labeled *GmmAttA1* or *GmmDef* probes overnight as described [2]. The *GAPDH* housekeeping gene was used to normalize RNA input. The abundance of mRNAs was determined using a Phosphorimager (PSI-Molecular Dynamics). One representative data set is shown from six independent replicates.

### Immunoblot analysis of procylin expression

*In vitro* maintained YTat1.1^WT^ or YTat1.1^EP^ PCF were used for immunoblotting. Parasites (5×10^3^) were boiled in Laemmli sample loading buffer and their proteins were separated by SDS-PAGE. After transfer to polyvinylidene difluoride membranes, procyclins were detected with EP specific mAb 274 [26] and GPEET specific mAb 5H3 [27], respectively. For analysis of procyclins *in vivo*, infections were initiated with YTat1.1^EP^ and YTat1.1^WT ^PCF and midguts from microscopically positive flies were dissected. Parasites were eluted from dissected midgut and proventriculus tissues by gentle homogenization and washed 5 times in serum-free SDM-79 medium. Cell numbers were determined with a hemocytometer and 2×10^3^ cells were used for immunoblot analysis.

### Parasite tagging and fly infections

The YTat1.1^WT^ and YTat1.1^EP^ PCF were transfected with pHD1034-GFP or pHD1034-RFP plasmid, respectively and the transformants were selected with 1 µg/ml puromycin [28]. The *in vitro* growth rate of each parasite line and parasite numbers in mixed cultures was measured over time by fluorescence microscopy using a hemocytometer. Parasite numbers in dissected and homogenized midgut extracts were similarly determined 14 days post acquisition. Infections were initiated either singly (10^6^ cells/ml) or as mixed infections (5×10^5 ^cells/ml of each parasite strain mixed). Although multiple experiments were performed the results from two representative experiments are shown.

### Impact of parasite infections on host mortality, fecundity and progeny

Female adults, 48 hours post emergence, received a single blood meal containing YTat1.1^WT^ or YTat1.1^EP^ parasites (10^5^ cells/ml) and those that fed were maintained on a normal blood meal diet. Mortality rates were determined for these two groups and an uninfected control group over a period of sixty days. To determine the effect of parasite infections on tsetse fecundity, all females were mated 96 hours post emergence and maintained in individual cages and monitored daily for larval deposition. Fly midguts were dissected and microscopically analyzed for infection status at the completion of the experiment.

Data were analyzed using SAS system v. 8.02 for Windows [29]. Student's *t*-tests or Mann Whitney *U*-tests were used to determine whether larval deposition time intervals significantly differed between trypanosome infected (YTat1.1^WT^ or YTat1.1^EP^) and control (both resistant and non-challenged) females. Student's *t*-tests were also employed to determine whether pupal weight and wing traits (length and width) significantly differed in the progeny. *F*-tests were applied to assess the homogeneity of variances.

### Quantitative reverse transcription-PCR (qRT-PCR) analysis of *GmmMGP*

Total RNA from eight YTat1.1^WT^ and YTat1.1^EP^ parasite infected 24 day old female flies and their age-matched normal controls were prepared. Following treatment with RNase-free Turbo DNase I (Ambion) the absence of DNA was confirmed by PCR amplification in a PCT-200 Peltier Thermal Cycler. One µg of total RNA was used for each sample for cDNA synthesis (Superscript II reverse transcriptase kit, Invitrogen, Carlsbad, CA). For qRT-PCR standard construction, inserts were cloned into the pGEM-T easy vector system (Promega, Madison, WI) [30]. Transcript quantification was performed on an iCycler Real-time detection system (Bio-Rad) and data were analyzed using software version 3.1. qRT-PCR for triplicate samples was performed using primers *GmmMGP*F 5′-CTGGACTCTTGACCCGTGAAC-3′ and *GmmMGP*R: 5′-GGGGAAGTGATGTTCCTTGA-3′ for 36 cycles at 58°C. For normalization, *G. m. morsitans tubulin* was amplified using the primer pair *GmmTub*F: 5′-GACCATGACGTGGATCACAG-3′ and *GmmTub*R: 5′-CCATTCCCACGTCTTCACTT-3′ for 36 cycles at 58°C.

## Results

### *T. b. rhodesiense* lines that exhibit different immunogenicity

**Figure 1:**
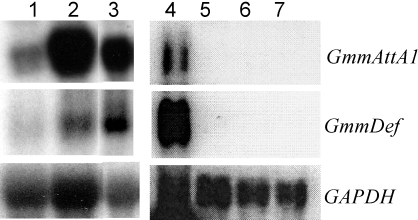
Expression of *attacin* and *defensin* in fat body. Lanes 1–3 flies receiving YTat1.1^WT^ trypanosome PCF supplemented blood meal; lane 1; 3 days post infective meal, lane 2; microscopically confirmed infected flies at day 10, and lane 3; microscopically confirmed parasite negative flies at day 10. Lane 4 fat body from flies 3 days post receiving *E. coli* injections. Lanes 5–7 flies receiving YTat1.1^EP^ PCF supplemented blood meal; lane 5; 3 days post infective meal, lane 6; microscopically confirmed infected flies at day 10, and lane 7; microscopically confirmed parasite negative flies at day 10.

Exposure of flies to wild type *T. b. rhodesiense* (Yale Trypanozoon antigenic type 1.1; YTat1.1^WT^) cells results in the upregulation of tsetse immune responses. For this analysis, we evaluated as immune markers the expression of two AMPs, *attacin* and *defensin,* which we had previously described from trypanosome-infected tsetse flies [2]. Infections with YTat1.1^WT^ trypanosomes activated the host immune system and induced the expression of *attacin* and *defensin* as early as 3 days post acquisition (Figure 1, lane 1). This heightened response persisted (when analyzed at day 10) in susceptible insects harboring midgut parasite infections (lane 2). The immune response was also evident in those resistant flies that lacked microscopically detectable parasite infections at day 10 (lane 3). Only later, at day 30, had the immune response of resistant flies subsided to normal levels, indicating the long lasting impact of parasite recognition on host immune stimulation (data not shown, [2]). By passaging procyclic culture forms at late log-phase, a cell line was selected and designated (YTat1.1^EP^). This parasite line did not induce tsetse AMP expression when analyzed at day 3 and day 10, following parasite acquisition (lanes 5–6, respectively). The phenotype of this trypanosome strain is described below.

### Procyclin surface coat variations

**Figure 2:**
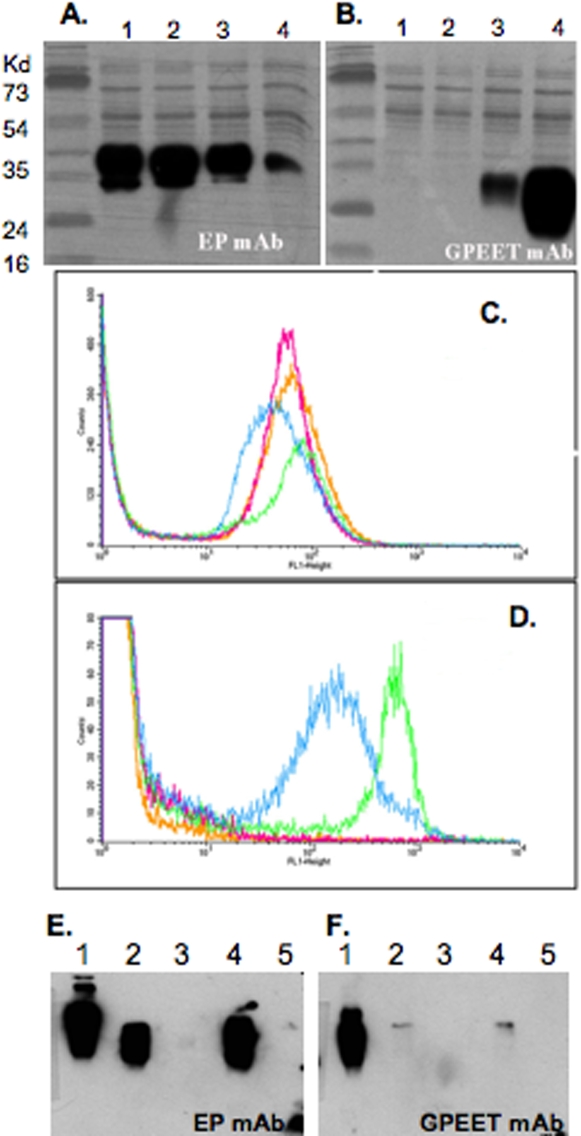
Immunoblot and flow cytometric analysis of procyclins expressed in YTat1.1^WT^ and YTat 1.1^EP^ procyclic culture forms. (A and B) Detection of EP procyclins with mAb 247 and GPEET procyclin with mAb 5H3, respectively. Lane 1: YTat 1.1^EP^ PCF (July 2003), Lane 2: YTat 1.1^EP^ PCF (Sept 2003), Lane 3: YTat1.1^WT^ PCF, Lane 4: *T. b. brucei* 427.01^WT^ PCF (positive control). Molecular weight markers are shown on the left. The autoluminograms are shown overlayed onto nigrosin stained membranes to show protein loadings. (C) Surface expression of EP on living PCF trypanosomes detected with mAb 247. (D) Surface expression of GPEET on living PCF trypanosomes detected with mAb 5H3. Blue lines: YTat 1.1^WT^ (Sept 2002), Green lines: YTat 1.1^WT^ (July 2004), Orange lines: YTat 1.1^EP^ (July 2003), Red lines: YTat 1.1^EP^ (Sept 2003). (E and F) Detection of EP and GPEET procyclins from (Lane 1) *in vitro* YTat1.1^WT^, (Lane 2) YTat1.1^WT^ obtained from midguts 12-days post infection acquisition, (Lane 3) YTat1.1^WT^ obtained from proventriculus 12-days post infection acquisition, (Lane 4) YTat1.1^EP^ obtained from midgut 12-days post infection acquisition and (Lane 5) YTat1.1^EP^ obtained from proventriculus 12-days post infection acquisition.

We first examined the expression of the cell surface procyclins on the immunogenic wild type parasites and the non-immunogenic strain that we had accidently selected in culture. We did this in order to understand the basis of the differential host immune activation, since the procyclic trypanosome surface coats are possible candidates for interaction with the fly's immune system. In *T. brucei* sspp., procyclins exist in several forms that are defined by the amino acid sequences of their C-terminal repeat domains carrying extensive glutamic acid-proline (EP) dipeptide repeats of differing lengths or pentapeptide repeats (GPEET). However, the expression of the procyclins is not static. It has been observed that the ratio of EP to GPEET procyclin expression *in vitro* can vary considerably between different parasite lines [27] or between different passages of the same culture [31] and that the composition of the coat may change in response to extracellular signals *in vitro* and during development *in vivo* [32]. Down-regulation of GPEET expression in trypanosomes has also been shown to be accelerated *in vitro* by hypoxia or be prevented by exogenous glycerol [32]. Procyclic culture forms of the non-immunogenic YTat1.1^EP^ parasites expressed EP and not GPEET procyclins, whereas the immunogenic YTat1.1^WT^ cells expressed both EP and GPEET procyclins, as determined using specific monoclonal antibodies (mAbs) by immunoblotting (Figure 2, panels A and B) and by flow cytometry (Figure 2, panels C and D). We do not know how our specific culture conditions caused the selection of the phenotype that exhibited decreased GPEET expression. However, the procyclin repertoire of the trypanosome lines we investigated was remarkably stable and did not change over several months of culture. Indeed, after being frozen for more than 6 months, thawed parasites grown in log-phase for a month exhibited the same phenotype (Figure 2C and D). Procyclic midgut trypanosomes purified from infected flies 12 days post parasite acquisition primarily expressed the EP procyclins with both parasite strains (Figure 2, D and F). Attempts to evaluate procyclin expression in epimastigotes in the proventriculus failed, likely due to the small number of parasites that could be obtained (Lane 3, Figure 2D and F). For these experiments, it is especially important to obtain pure parasite preparations given the cross reactivity EP monoclonal antibody exhibits with the unrelated tsetse EP proteins [33]. Nevertheless, these results suggest that *in vivo* YTat1.1^WT^ parasite undergo a similar differentiation process as YTat1.1^EP^ with respect to the expression of procyclins. It is of interest that the non-immunogenic trypanosomes originally fed to tsetse did not express GPEET whereas the immunogenic wild type parasites did.

### Fly infections with YTat1.1^WT^ and YTat1.1^EP^

**Figure 3:**
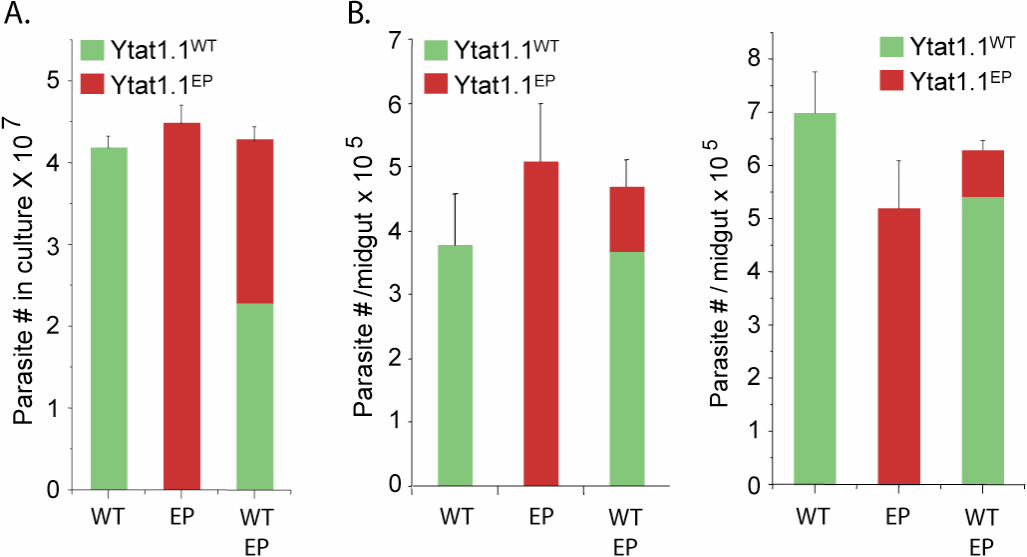
Growth dynamics of YTat1.1^WT^ and YTat1.1^EP^ trypanosomes *in vitro* and *in vivo*. (A) *In vitro* culture densities (average of three separate experiments) maintained alone or as mixed cultures. Red bars denote RFP-YTat1.1^EP^ and green bars denote GFP-YTat1.1^WT^. (B) Concentration of parasites within parasite infected midgut either with one strain alone or as mixed infections. Each group had 6–8 infected midguts and results from two independent experiments are shown.

Both YTat1.1^WT^ and YTat1.1^EP^ parasites had similar growth rates with a doubling time of 12±0.15 hours under *in vitro* cultivation conditions. The parasite lines were labeled with green fluorescent protein (GFP-YTat1.1^WT^) or red fluorescent protein (RFP-YTat1.1^EP^), respectively, to allow visual discrimination in mixed infection experiments. There was no statistically significant difference between the numbers of red and green trypanosomes in *in vitro* cultures that were initiated with single phenotype or mixed parasites (Figure 3A).

Prevalence of midgut infections with trypanosomes YTat1.1^WT^ and YTat1.1^EP^ were compared (Table 1), but showed no significant differences (p = 0.726. No significant difference in midgut infection intensity (parasite numbers) was found two weeks post infection acquisition with either parasite line (Figure 3B). However, when fly infections were established by feeding equal numbers of a mixture of the tagged parasites, a significantly higher number of YTat1.1^WT^ cells were seen in established midgut infections (Figure 3B). Prior studies have shown that YTat1.1 cells exhibit high virulence in the mammalian host [34]. Since the YTat1.1 cells that we used have lost the ability to establish salivary gland infections in the fly, we were unable to compare the transmission potential of the two parasite lines and the potential virulence of YTat1.1^EP^ for mammalian hosts. We thus limited our analysis to the impact of midgut parasite infections on tsetse fecundity and resulting population structure.

**Table 1:** Midgut infection prevalence in flies challenged with either YTat1.1^WT^ or YTat1.1^EP^.

Parasite line	N, pooled*	N, Infected	% Infection prevalence (SD)
Ytat1.1^WT^	81	6	7.1 (1.4)
Ytat1.1^EP^	93	8	8.1 (2.1)

*: Three independent experiments were combined, p = 0.7256, SD:Standard Deviation.

**Table 2:** Larval deposition intervals of YTat1.1^WT^ (bold) and YTat1.1^EP^ infected and uninfected females.

	Mean±S.E. (N)	P-value
**To 1^st^ deposition;**		
**Uninfected** *	**26.4±1.0 (36)**	**P = 0.0006**
**YTat1.1^WT^ infected**	**31.8±1.1 (45)**	
**To 2^nd^ deposition;**		
**Uninfected** *	**13.3±0.9 (30)**	**P = 0.036**
**YTat1.1^WT^ infected**	**17.0±1.5 (40)**	
**To 3^rd^ deposition;**		
**Uninfected** *	**13.4±0.8 (17)**	**P = 0.029**
**YTat1.1^WT^ infected**	**17.6±1.5 (24)**	
To 1^st^ deposition;		
Uninfected*	23.9±5 (48)	P = 0.78
YTat1.1^EP^ infected	23.6±2 (14)	
To 2^nd^ deposition;		
Uninfected*	11.1±3.0 (37)	P = 0.512
YTat .1^EP^infected	11.2±3.2 (14)	
To 3^rd^ deposition;		
Uninfected*	10.4±2.1 (25)	P = 0.612
YTat1.1^EP ^infected	10.1±0.7 (7)	

*: Control females and challenged but uninfected (resistant) females were combined because of the lack of significance between these two (P<0.05).

### Fitness cost of trypanosome infections

We compared the mortality and fecundity of flies infected with each parasite line for up to 60 days. There was no significant difference in mortality rates between the YTat1.1^WT^ parasite exposed group and the age-matched, unchallenged control group (30.6% versus 30%, respectively). We next compared the fecundity of fertile females infected with YTat1.1^WT^ and YTat1.1^EP^ trypanosomes to age matched, uninfected controls (Table 2). Females were monitored daily for larval deposition through their initial three reproductive cycles. The infection status of the females was microscopically confirmed by examination of dissected midguts at the conclusion of the experiment. Our experiments with YTat1.1^WT^ and YTat1.1^EP^ parasites were conducted during 2002 and 2006, respectively. The difference in the larval deposition periods observed between these experiments can reflect environmental variations such as temperature and humidity conditions in the insectary. Hence, each experimental group was compared to its age-matched control group subjected to the same environmental conditions. For final analysis, the control group consisted of the nonchallenged and challenged but uninfected (resistant) females because of the lack of any significant differences between these two groups (P>0.05). All three larval deposition periods of flies infected with YTat1.1^WT^ parasites were found to be significantly longer than those of the corresponding uninfected controls, while YTat1.1^EP^ infected females had similar larval deposition periods to those of their control group.

### Effect of trypanosome infections on host gene expression

**Figure 4:**
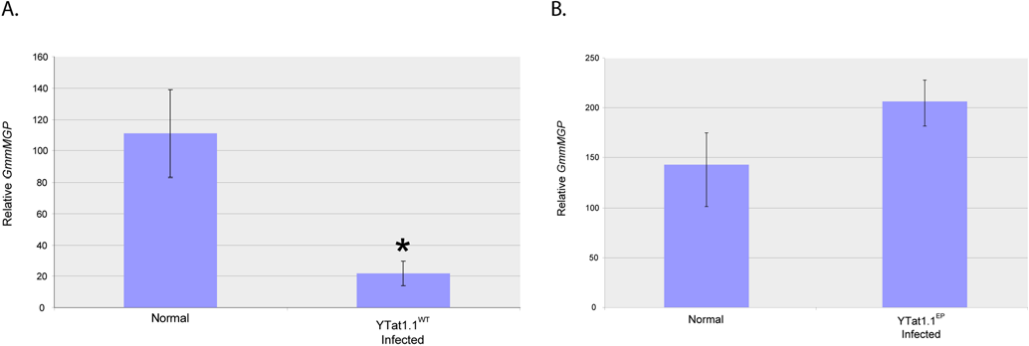
qRT-PCR expression analysis of the major milk gland protein *GmmMGP* from YTat1.1^WT^ infected and age-matched uninfected controls (A) and YTat1.1^EP^ infected and age-matched uninfected controls (B). The bars denote standard error values observed among eight individuals tested in each group. The * denotes significant difference from control P<0.05.

We also evaluated the expression of tsetse larvagenesis associated protein, (milk gland protein; *GmmMGP*). GmmMGP is an abundant milk protein synthesized by the female accessory gland tissue [35] and supplied to the developing intrauterine progeny in the “milk” secretion [36]. Fertile females infected with the immunogenic YTat1.1^WT^ parasite strain exhibited significantly decreased expression levels of *GmmMGP* in comparison to uninfected age-matched control females (Figure 4A). A similar reduction in *GmmMGP* was not observed in flies infected with YTat1.1EP parasite strain (Figure 4B), suggesting that the delayed larvagenesis process in immune stimulated flies may result from the decreased expression of the *GmmMGP* protein, which is necessary for larval growth.

### Effect of parasite infections on fitness of tsetse progeny

In addition to the cost on direct life-history traits, we obtained morphological data reflective of adult fitness from three sequential progeny produced by mothers infected with YTat1.1^WT^ parasites and compared these to an uninfected cohort (Table S1). There was no difference in pupal weight or in hatch rate (percent pupae that successfully emerged) between the two groups of progeny. In addition, there were no significant differences in wing width and length of the hatched progeny between the two groups. Based on morphological findings, delayed larvagenesis does not apparently result in loss of fitness of future progeny despite reducing the reproductive output of the mother.

### Calculating fecundity differences

We performed a mathematical analysis to translate the empirically measured times between larva depositions (Table 2) into the relative differences in fecundity of tsetse infected with the immunogenic versus the non-immunogenic trypanosome strains. In our analysis, we assumed that a proportion *s*_P_ of deposited larva survive pupation to become adults, while the expected duration of the pupation is *l*_P_. We let *m* be the proportion of offspring that are female. We also assumed a constant adult death rate, μ_A_, resulting in the expected duration of the adult stage 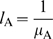. The time to first deposition was denoted *t*_1_ and the time between subsequent depositions *t*_2_. Thus, the second deposition occurs at time *t*_1_+*t*_2_, the third deposition at *t*_1_+2*t*_2_, and so on. The expected total number of female offspring of a female parent over her lifetime is then
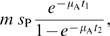
(1)which, when divided by the expected life span, *l*_P_+*l*_A_, gives the reproductive rate
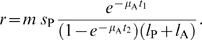
(2)


Reproductive rates in the absence of trypanosome infection, *r*_U_, and in the presence of infection, *r*_I_, are related by
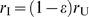
(3)so that ε is the relative fecundity cost of infection. Then
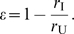
(4)


Taking *t*_1_ to be the time to first deposition from our empirical measurements (Table 2), *t*_2_ to be given by the mean of the times to the second and third depositions (Table 2), *s*_P_ = 0.82, *l*_P_ = 31.4 days, and μ_A_ = 0.0253 day^−1^ [37] gives ε = 0.3. That is, trypanosome infection causes a reduction in fecundity of approximately 30%.

### Differential population growth of tsetse flies

We next converted the fecundity differences calculated at the level of the individual fly into differences for tsetse population growth depending on whether or not the tsetse are infected. The population growth rate, *r*, is the root of the Euler–Lotka equation [38],[39].
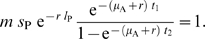
(5)For the parameter values above and 50% of offspring being female (*m* = 1/2), equation (5) gives *r*_U_ = 0.0008 day^−1^ for uninfected tsetse and *r*_I_ = −0.0025 day^−1^ for a population composed entirely of infected tsetse. Thus, for uninfected tsetse, *r*_U_ is slightly positive, indicative of the slow viviparous reproduction of tsetse, while *r*_I_ is negative, which would lead to population collapse if all tsetse were infected.

If the probability of surviving the pupal period is decreased to *s*_P_ = 0.82 ([37] for *G. palpalis gambiensis*), the growth rate is negative, *r*_U_ = −0.0012 day^−1^, while for infected tsetse, *r*_I_ = −0.0044. With *s*_P_ = 1, an increased death rate of μ_A_ = 0.030 day^−1^ has an even stronger effect on the basic reproduction number and growth rate, *r*_U_ = −0.00046 day^−1^ and *r*_I_ = −0.0082 day^−1^.

**Figure 5:**
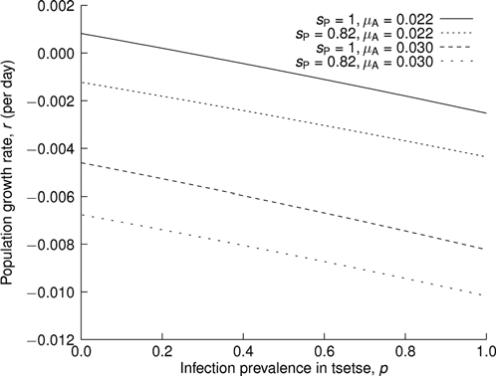
The relationship between population growth rate and prevalence of immunogenic trypanosome strain for different pupal survival (*s*_P_) and adult mortality rates (μ_A_). In all cases, the curves are well approximated by lines with slope between about −0.0031 and −0.0036 per day, so that a 10% increase in prevalence corresponds to a 0.031–0.036 per day decrease in population growth rate. The units of μ_A_ are per day.

For a varying level of infection, if we assume no vertical transmission, the population growth rate for the overall population is given by
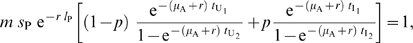
(6)where *p* is the prevalence of antigenic virulent trypanosome infection in tsetse. With *s*_P_ = 1 and μ_A_ = 0.022 day^−1^, at around *p* = 0.26, *r* = 0. For infection prevalence below 26%, the tsetse population continues to grow, while prevalence above 26% gives declining tsetse populations (Figure 5). The relationship between population growth rate and infection prevalence is monotone but not linear. The prevalence of trypanosomiasis would be expected to be reduced with a decline in the vector tsetse population. These modeling results are not intended to be precise quantitative predictions, but indicate likely qualitative dynamics of the interaction between tsetse and trypanosomes. Thus, the main conclusion that infection with the immunogenic parasite strain has the potential to suppress the tsetse population should apply broadly. However, the precise prevalence of trypanosomiasis that result in negative tsetse population growth depends on the exact parameter values, which may vary seasonally and spatially.

## Discussion

We studied the tsetse-trypanosome system to evaluate the molecular and physiological aspects of host-parasite interactions and the consequences of parasite infections on host fecundity. Two trypanosome strains that differentially activate host immunity were employed to assess the cost of the ability to clear parasite infections (resistance) and the cost of midgut parasite infections in susceptible flies. In contrast to the dogma that expression of parasite resistance traits incurs a fitness cost to the insect host, we observed no significant fecundity cost associated with trypanosome resistance in laboratory reared tsetse. However, activation of tsetse immune responses by infecting flies with immunogenic trypanosomes reduced host reproductive output while infections with non-immunogenic trypanosomes did not.

It is unusual that we did not detect a fecundity cost associated with parasite resistance in tsetse. This observation, that differs from what is seen in mosquitoes, may reflect the viviparous reproductive biology of tsetse where investments in reproductive output begin significantly later and continue throughout the lifetime of adult female flies. Female tsetse develop a single oocyte per gonotrophic cycle. Following ovulation and fertilization, the embryo develops within the uterus and hatches into a first instar larva, which molts through two more instars before being deposited as a fully developed larva which quickly pupates in the soil. From this point on, there is a continuous investment in nurturing the single larva produced one at a time, approximately every ten days, through the remainder of the female lifespan. This differs dramatically from mosquitoes, which have a high reproductive output as young adults and hence may be more sensitive to perturbations early in life. We cannot rule out however that our *in vitro* experiments conducted under uniform environmental conditions and ample nutritional supply may have skewed any potential fecundity cost that flies may experience upon expression of parasite resistance in the wild.

Reduced host fecundity has been observed in various systems with parasitized insects (reviewed in Hurd, 2003). Our result is the first demonstration of a significant loss of fecundity in parasitized tsetse but only when infected with an obviously immunogenic trypanosome line. Loss of fecundity in tsetse may arise from the cost of prolonged immune activation since a similar fitness cost was not observed in flies infected with the non-immunogenic parasites. Our results suggest that the decreased expression of milk gland protein *GmmMGP*, the most abundant protein product in the “milk” secretion of modified accessory glands, may cause the observed delayed larvagenesis process [36],[40]. We recently noted that in addition to *GmmMGP*, *transferrin*, which is also expressed in the female milk gland organ and is transported into the developing larva is down regulated by YTat1.1^WT^ infections [41]. A similar reduction in the abundance of vitellogenin (Vg) mRNA and the titer of circulating Vg in the hemolymph has been reported in plasmodium infected mosquitoes during early oocyst development [42]. Later in the infection process, changes in the ovarian follicular epithelium have also been associated with a decrease in Vg uptake by the ovary and may also result in reduced hormone ecdysone production, which is needed for the transcriptional regulation of *Vg* expression [43]. Our results suggest several avenues for future research. It remains to be seen whether the regulation of host gene expression could result from an adaptive strategy that has evolved in response to parasitism, or that it reflects the manipulation of the host insect by the parasites. The regulation of milk-gland protein and transferrin transcription remains to be described, but may similarly be subject to hormonal regulation, which in turn may be influenced by parasite infections.

One potential difference we identified between the trypanosome strains used to infect tsetse was in their EP and GPEET procyclin compositions. Under our culture conditions, the immunogenic YTAT^WT ^line expressed both major forms of procyclin, while the YTAT^EP^ line only expressed EP procyclins. Procyclin epitopes analyzed from parasites obtained from midgut infections indicated that both lines expressed the EP isoforms later in the infection process, similar to previous reports [44]. It is possible that the highly phosphorylated GPEET molecules present on YTAT^WT^ procyclic culture form parasites, upon acquisition in the blood meal, are recognized by the host's immune system early in the infection process and elicit the immune activation we observed in our study. Supporting this idea, when beads carrying different surface charges were introduced into mosquitoes, different physiological networks were induced resulting in varied vector cost outcomes [45]. It is possible that as a consequence of host-parasite co-evolutionary dynamics, trypanosomes have evolved to down regulate the expression of GPEET in order to avoid inducing host resistance mechanisms. It is entirely possible however, that variations in parasite molecules other than procyclins may be responsible for the observed host immune outcome. The expression of GPEET alone in the early infection process cannot explain the sustained induction of host immune effectors in the case of infections with the immunogenic line since this line also switches to express predominantly the EP procyclins in late midgut infections. As molecular data on tsetse immunity are accumulating, it would be of interest to conduct a global expression analysis of both RNA and protein using the two parasite lines to gain a broader appreciation of the host-parasite interactions in the tsetse system [46].

Multiple vector immune components are likely to play a role in parasite transmission and resistance phenotype. Although our previous results have identified an important role for antimicrobial peptides in parasite establishment in tsetse, lack of AMP expression does not result in greater parasite infection prevalence for YTAT^EP^. It is possible that other trypanocidal effectors, such as lectins [14]–[17] and antioxidant activity in the midgut milieu at the time of parasite acquisition that are necessary for resistance. Alternatively, cascades in immunity pathways could result in the synthesis of different trypanolytic immune effectors, which are uncharacterized at the present time. The similar midgut infection intensities observed with both trypanosome lines may also suggest general regulatory processes whereby homeostasis of parasite density is maintained to prevent excessive harm on host physiology. Our mixed infection experiments *in vivo* however showed that the immunogenic parasites outcompeted the non-immunogenic parasites in midguts. It is plausible that tsetse immune products, in particular antimicrobial peptides, may have varying trypanolytic effect on parasites with initial differences in surface protein composition. It is then possible that infections with the immunogenic parasites, invoking host immune responses, could prevent the transmission of other strains that may be more sensitive to the host immune products.

The prevalence of tsetse infections reported with *T. brucei* sspp. in the wild is low, typically in the order of 1–3% [47]–[49], suggesting the potential for only a small impact on the whole tsetse population. However, the prevalence of infections with other trypanosome species such as *T. congolense* and *T. vivax*, can be significantly higher, for example 28% reported in Burkina Faso [50] and 24% in Tanzania [51]. Furthermore infections with mixed trypanosome species are common, comprising over 30% of infections reported in natural populations [52]–[54]. Our modeling results suggest that these higher infection rates could have substantial effect on the tsetse population dynamics if the presence of these parasite species were to cause similar reproductive delays. Such reproductive delays caused by immunogenic trypanosome strains could lead to a reduction in tsetse population size and consequently to less transmission of parasites to vertebrate hosts. In turn, the resulting reduced trypanosome prevalence in vertebrates would lead to a lower prevalence in tsetse and a corresponding rebound of the tsetse population until an equilibrium of trypanosome prevalence in tsetse and vertebrate hosts is reached at lower levels than would occur with an avirulent strain. The mechanisms of tsetse population regulation are not well understood [55], so the magnitude of the impact of trypanosome-induced fecundity delays on trypanosome infections in vertebrates is difficult to predict.

Assuming that both parasite lines we investigated have equal probability for maturation in the salivary glands for mammalian transmission, our co-infection dynamics would favor the spread of the immunogenic phenotype. Reducing its tsetse host's fitness is evolutionarily disadvantageous to the trypanosome, but only weakly compared to the possible benefits of increased virulence. Interestingly, YTat1.1^WT^ is known to be highly virulent in the mammalian host, but we do not have a virulence phenotype for YTat1.1^EP^ since we cannot obtain salivary gland mammalian transmissible infections with this line. Trade-offs between virulence and parasite transmissibility can maintain virulence, as can competition within a host between multiple strains of a parasite [56]. Co-evolution between pathogen and host could lead to more complicated dynamics, from the emergence of mutualism to an escalating cycle of virulence and host resistance. In fact, a field study demonstrated that the resistance of *Anopheline* mosquitoes to *Plasmodium* parasite development varied considerably between different combinations of parasite and vector isolates, suggesting that there are specific compatibilities between the insect and parasite genotypes [57]. If there is no cost of resistance against virulent trypanosomes, we would expect resistance to increase until the “virulent” trypanosome is driven extinct. In contrast, the “avirulent” trypanosome would not impose selection on its tsetse host, and would persist. If there is a cost of resistance however, there would be an equilibrium proportion of resistance genotype at which the cost of resistance is balanced by the cost and prevalence of virulent infection. In this case, if the avirulent trypanosome strain without any fecundity cost emerged and spread, genetically susceptible tsetse would be expected to completely replace resistant tsetse. Simultaneously, disease prevalence in both tsetse and mammalian hosts would rise. Future experiments will need to be conducted with flies in the field to understand the impact of natural parasite infections on host immunity, and the potential burden of host immune activation on fecundity in order to relate to disease epidemiology. Our laboratory findings coupled with our modeling studies now provide a framework to investigate the status of co-infections, host immune activation processes, fecundity outcomes, transmission dynamics and host virulence phenotypes in natural tsetse-trypanosome populations.

## Supporting Information

Table S1 Progeny fitness parameters for male and female [shaded] tsetse uninfected* and infected with YTat1.1^WT^.
* Progeny of control and uninfected (resistant) females were combined in all three observations because of the lack of any significant differences (P<0.05). The weight of pupae at time of deposition, eclosion rate of puparia deposited and the wing traits (width and length) of viable offspring adult were measured for progeny deposited by YTat1.1^WT^ fed flies and the uninfected control group. Wing length was measured from the wing base to the distal end of the radial cell, while wing width was determined from the distal end of the alula to the costal vein (Comstock-Needham system). Measurements were accurate to within 0.02 mm.
(0.04 MB DOC)
