# Supplementary material for: Infections with Immunogenic Trypanosomes Reduce Tsetse Reproductive Fitness: Potential Impact of Different Parasite Strains on Vector Population Structure
Source: PLoS Negl Trop Dis. 2008 Mar 12;2(3):e192. doi: 10.1371/journal.pntd.0000192 (PMC2265429; doi:10.1371/journal.pntd.0000192)
Supplement: Table S1 — Progeny fitness parameters for male and female [shaded] tsetse uninfected* and infected with YTat1.1WT. * Progeny of control and uninfected (resistant) females were combined in all three observations because of the lack of any significant differences (P<0.05). The weight of pupae at time of deposition, eclosion rate of puparia deposited and the wing traits (width and length) of viable offspring adult were measured for progeny deposited by YTat1.1WT fed flies and the uninfected control group. Wing length was measured from the wing base to the distal end of the radial cell, while wing width was determined from the distal end of the alula to the costal vein (Comstock-Needham system). Measurements were accurate to within 0.02 mm. (0.04 MB DOC) [file pntd.0000192.s001.doc]

Table S1. Progeny fitness parameters for male and female [shaded] tsetse uninfected* and infected with.YTat1.1^WT^.

Mean ^+^S.E. (sample size) P-value

| Pupal weight (g)  Uninfected*  YTat1.1^WT^ infected | 0.024 ^+^  0.0004 (37)  0.024 ^+^  0.0028 (92) | P=0.975 |
| --- | --- | --- |
| Female wing length (cm)  Uninfected*  YTat1.1^WT^ infected | 0.77 ^+^  0.015 (10)  0.796 ^+^  0.0038 (31) | P=0.12 |
| Male wing length (cm);  Uninfected*  YTat1.1^WT^ infected | 0.727 ^+^  0.0156 (11)  0.71 ^+^  0.006 (38) | P=0.26 |

* Progeny of control and uninfected (resistant) females were combined in all three observations because of the lack of any significant differences (P<0.05).

The weight of pupae at time of deposition, eclosion rate of puparia deposited and the wing traits (width and length) of viable offspring adult were measured for progeny deposited by YTat1.1^WT^ fed flies and the uninfected control group. Wing length was measured from the wing base to the distal end of the radial cell, while wing width was determined from the distal end of the alula to the costal vein (Comstock-Needham system). Measurements were accurate to within 0.02 mm.
